# Supplementary material for: Is brain imaging necessary for febrile elderly patients with altered mental status? A retrospective multicenter study
Source: PLoS One. 2020 Jul 30;15(7):e0236763. doi: 10.1371/journal.pone.0236763 (PMC7392262; doi:10.1371/journal.pone.0236763)
Supplement: Attachment — Submitted filename: Response_to_reviewers.docx [file pone.0236763.s002.docx]

Dear Reviewer

Mehmet Ali Aslaner

We appreciate the very kind comments by reviewer, which greatly improved our manuscript. Our replies to the comments and amended manuscript are as follows:

**Reviewer:
1. Abstract:**

**• What does it mean “Pneumonia and urinary tract infection were the most common in controls.” Authors wanted to mean that “The most common diagnoses in these patients admitted to the emergency department were pneumonia and urinary tract infection.”**

**Reply:** Thank you for your kind comments. As your comments, we revised the sentence you pointed out to make it easier to understand. We wanted to mean “The most common diagnoses in patients admitted to the emergency department were intracranial hemorrhage and ischemic stroke for ABI, and pneumonia and urinary tract infection for non-ABI”

Page. 2, Line 13-15

In all, 285 patients were enrolled, and 47 (16.49%) showed ABI. Intracranial hemorrhage and ischemic stroke were most common. Pneumonia and urinary tract infection were the most common in controls.

🡺

In all, 285 patients were enrolled, and 47 (16.49%) showed ABI. The most common diagnoses in patients admitted to the emergency department were intracranial hemorrhage and ischemic stroke for ABI, and pneumonia and urinary tract infection for non-ABI.

**• Authors should state “OR” and “95% CI” in the result when specifying logistic regression analysis.**

**Reply:** Thank you for the point. As you suggested, we stated “OR” and “95% CI” in the result to specify logistic regression analysis.

Page. 2, Line 15-20

In multivariate logistic regression analyses, higher systolic blood pressure (odds ratio, 1.017; 95% confidence interval, 1.006–1.028), lower body temperature (0.578, 0.375–0.892), the presence of lateralizing sign (45.676, 5.015–416.025), and lower Glasgow Coma Scale (0.718, 0.617–0.837) were significantly associated with ABI.

🡺

In multivariate logistic regression analyses, higher systolic blood pressure (odds ratio [OR], 1.017; 95% confidence interval [CI], 1.006–1.028), lower body temperature (OR, 0.578; 95% CI, 0.375–0.892), the presence of lateralizing sign (OR, 45.676; 95% CI, 5.015–416.025), and lower Glasgow Coma Scale (OR, 0.718; 95% CI, 0.617–0.837) were significantly associated with

**2. Materials and Methods:**

**• AMS definition was poor, so authors should elaborate the AMS criteria.**

**Reply:** Thank you. As you pointed out, we agree that the definition of AMS was poor in our manuscript. We changed the term ‘decreased consciousness’ to ‘altered consciousness’, and clarified the definition of AMS.

Page 5, line 7-9

AMS was defined as a Glasgow Coma Scale (GCS) of 14 or less, or decreased consciousness on chief complaint

🡺

AMS was defined as a state of Glasgow Coma Scale (GCS) of 14 or less, or new onset of altered consciousness such as drowsiness, behavior change, unresponsiveness, disorientation or confusion [9].

**• In data collection, authors stated that “Two emergency doctors reviewed the results”. This is one of the most important places in this study. How competent were the two doctors? Were the AMS diagnoses clear in all patients? These and similar issues should be more detailed.**

**Reply:** Thank you for the good point. We should have made it clearer. The doctors who reviewed the results were described rather insufficiently as it was only stated as “two emergency doctors”, so we specified them as “emergency physicians” to show that the two doctors had sufficient clinical experience.

We understand your concern about whether AMS diagnoses were clear in all patients. We also agree that the diagnosis criteria of AMS was described unclear in our manuscript, so we described the criteria more specifically so it can be understood more clearly. Thus, in this way, we could diagnose AMS in all the patients clearly following this criteria (though described rather poorly in the previous manuscript). Sorry that we presented ambiguous description and made you misunderstand.

Page 6, line 1-2

Two emergency doctors reviewed the results.

🡺

All patient's medical records and results were reviewed by two emergency physicians, who had more than 10-years of clinical experience.

**• It was stated that “Fever was defined as a case where the patient's body temperature (BT) was 38 degrees or more at the time of visit hospital or at home.” Can home fever measurement be reliable for a retrospective study**?

**Reply:** Thank you. We actually included ‘home fever’ in the first place because we found some papers that included ‘home fever’ when defining fever. (related studies are presented below) However, as you pointed out, we agree that there is a possibility of unreliability, so we added this in the limitations section. Thank you for pointing out what we missed.

Behrman, R. E., Meyers, B. R., Mendelson, M. H., Sacks, H. S., & Hirschman, S. Z. Central nervous system infections in the elderly. Arch Intern Med. 1989;149(7), 1596-1599.

Ko, H. F., Tsui, S. S., Tse, J. W., Kwong, W. Y., Chan, O. Y., & Wong, G. C. Improving the emergency department management of post-chemotherapy sepsis in haematological malignancy patients. Hong Kong Med J. 2015;21(1), 10-15.

Page 14, Line 23-24

Third, we included the cases who had fever at home in the study population [21, 22], but fever measured at home may not be reliable.

Page 18, Line 23-27

21.	Behrman, R. E., Meyers, B. R., Mendelson, M. H., Sacks, H. S., & Hirschman, S. Z. Central nervous system infections in the elderly. Arch Intern Med. 1989;149(7), 1596-1599.

22.	Ko, H. F., Tsui, S. S., Tse, J. W., Kwong, W. Y., Chan, O. Y., & Wong, G. C. Improving the emergency department management of post-chemotherapy sepsis in haematological malignancy patients. Hong Kong Med J. 2015;21(1), 10-15.

**3. Result:**

**• In table 1, what does “witness” mean? It should be stated in the method or elsewhere.**

**Reply:** Thank you for the point. We may have caused confusion because we misused the word ‘witness’ instead of ‘witnessed’. We should have used ‘witnessed’. We also missed out definition of ‘witnessed’ in the manuscript. We defined ‘witnessed’ using the the definition of ‘witnessed’ in Standard Utstein-style guideline and we added this information newly in the method section.

Page 5, Line 20-21

The onset of AMS was classified into acute (less than 24 h), subacute (1–7 days), and chronic (>7 days) [9]. All patients received physical and neurological examinations, conducted by a senior resident or emergency medicine specialist

🡺

The onset of AMS was classified into acute (less than 24 h), subacute (1–7 days), and chronic (>7 days) [9]. Witnessed is defined as AMS that is seen or heard by another people when it happened [11]. All patients received physical and neurological examinations, conducted by a senior resident or emergency medicine specialist.

Page 17, Line 24 – Page 18, Line 4

11. Jacobs I, Nadkarni V, Bahr J, Berg RA, Billi JE, Bossaert L, et al. Cardiac arrest and cardiopulmonary resuscitation outcome reports: update and simplification of the Utstein templates for resuscitation registries: a statement for healthcare professionals from a task force of the International Liaison Committee on Resuscitation (American Heart Association, European Resuscitation Council, Australian Resuscitation Council, New Zealand Resuscitation Council, Heart and Stroke Foundation of Canada, InterAmerican Heart Foundation, Resuscitation Councils of Southern Africa). Circulation. 2004;110(21):3385-97.
